# *Paenibacillus polymyxa* NSY50 suppresses *Fusarium* wilt in cucumbers by regulating the rhizospheric microbial community

**DOI:** 10.1038/srep41234

**Published:** 2017-02-13

**Authors:** Lu Shi, Nanshan Du, Sheng Shu, Jin Sun, Shuzhan Li, Shirong Guo

**Affiliations:** 1 Key Laboratory of Southern Vegetable Crop Genetic Improvement in Ministry of Agriculture, College of Horticulture, Nanjing Agricultural University, Nanjing 210095, People’s Republic of China; 2 Suqian Academy of Protected Horticulture, Nanjing Agricultural University, Suqian, China

## Abstract

*Paenibacillus polymyxa (P. polymyxa*) NSY50, isolated from vinegar residue substrate, suppresses the growth of *Fusarium oxysporum* in the cucumber rhizosphere and protects the host plant from pathogen invasion. The aim of the present study was to evaluate the effects of NSY50 application on cucumber growth, soil properties and composition of the rhizospheric soil microbial community after exposure to *Fusarium oxysporum.* Bacterial and fungal communities were investigated by Illumina sequencing of the 16S rRNA gene and the internal transcribed spacer (ITS) regions (ITS1 and ITS2). The results showed that NSY50 effectively reduced the incidence of *Fusarium* wilt (56.4%) by altering the soil physico-chemical properties (e.g., pH, C_mic_, R_mic_, total N and C_org_) and enzyme activities, especially of urease and β-glucosidase, which were significantly increased by 2.25- and 2.64-fold, respectively, relative to the pathogen treatment condition. More specifically, NSY50 application reduced the abundance of *Fusarium* and promoted potentially beneficial groups, including the *Bacillus, Actinobacteria, Streptomyces, Actinospica, Catenulispora* and *Pseudomonas* genera. Thus, our results suggest that NSY50 application can improve soil properties, shift the microbial community by increasing beneficial strains and decreasing pathogen colonization in the cucumber rhizosphere, and reduce the occurrence of cucumber *Fusarium* wilt, thereby promoting cucumber growth.

Cucumber *Fusarium* wilt, caused by *Fusarium oxysporum* f. sp. *cucumerinum* (FOC), is a typical soil-borne fungal disease that leads to decreased cucumber production and significant economic losses^1^. The symptoms of *Fusarium* wilt are yellowing and necrosis of foliage, followed by foliar wilting and dark brown discoloration of the roots, even death of the entire plant as the pathogen invades the vascular system of the cucumber^2^. Plant death typically occurs in a few days or weeks. Traditional management methods, including the use of resistant cultivars, seedling grafting, crop rotation, and chemical strategies, have been suggested to control *Fusarium* wilt, but these approaches are not economical, reliable or environmentally friendly^3^,^4^,^5^,^6^.

To date, biological control has been recognized as an effective and sustainable approach to combat *Fusarium* wilt^7^. It is widely accepted that certain strains of rhizospheric bacteria and fungi, termed biocontrol agents (BCAs), can protect plants from soil-borne pathogens and improve plant growth. Amongst the BCAs, the best reported bacterial genera are *Pseudomonas* spp.^8^,^9^ and *Bacillus* spp.^10^,^11^,^12^, which have shown potential as BCAs against different fungal pathogens. BCAs can suppress disease and increase crop productivity, which are influenced by antibiosis, nutrient and biological niche competition, heavy parasitism, and the induction of resistance^13^. In recent years, *Streptomyces albospinus* CT205 has been identified as a potential biological control agent to suppress *Fusarium* wilt in cucumber plants^14^. Abdallah *et al*.^15^ also found that *Fusarium* wilt severity was significantly decreased following exposed to *Bacillus* sp. str. SV101 and SV104 in pathogen-challenged tomato plants. In another study, *P. polymyxa* strains were shown to cause antibiosis and produce polymyxins, colistin and hydrolytic enzymes, which play important roles in the biocontrol of plant pathogens^16^,^17^,^18^. Furthermore, the microbial community of the rhizosphere is primary factor determining plant health^19^, and also thought to be distinctly important to the biological, chemical and physical processes that are essential to maintain a healthy and stable microenvironment and to successfully suppress various diseases^20^,^21^,^22^. In turn, the soil enzymatic activities, plant species, and soil type also influence the composition of the microbial community, contributing to plant disease suppression^23^,^24^,^25^,^26^.

The diversity of microbes can be evaluated by conventional isolation cultures and plate counting, phospholipid fatty acid (PLFA) analysis and denaturing gradient gel electrophoresis (DGGE)^27^,^28^. However, culture-based methods are time-consuming, and the sequencing depth needed to identify more microbes cannot be achieved by using these methods. Recently, illumina Miseq PE300 has been applied as a next-generation sequencing (NGS) method for the in-depth analysis of soil microbial community structures^29^. The 16S rRNA gene and the internal transcribed spacer (ITS) region are now widely used to analyse soil bacterial and fungal communities^30^,^31^,^32^,^33^, respectively. This approach has provided insights into ecological processes and the soil microbial community. To the best of our knowledge, the microbial community of cucumbers challenged with *P. polymyxa* NSY50 has not yet been assessed using high-throughput sequencing.

*P. polymyxa* NSY50 was originally isolated from vinegar residue substrate (VRS). VRS is a novel organic matrix for gardening that is produced by the vinegar-making industry. In our previous study, VRS was shown to effectively control cucumber *Fusarium* wilt^34^. In addition, a dual-culture antagonistic bioassay showed that NSY50 has broad-spectrum resistance. Therefore, the objectives of this study were (1) to evaluate the effects of NSY50 on the suppression of FOC in cucumbers, (2) to compare the differences in the composition of the rhizospheric microbial community after challenge with NSY50 and FOC using Illumina sequencing technology, and (3) to further illustrate the mechanisms of soil-borne disease suppression.

## Results

### Disease incidence and plant growth

**Figure 1:**
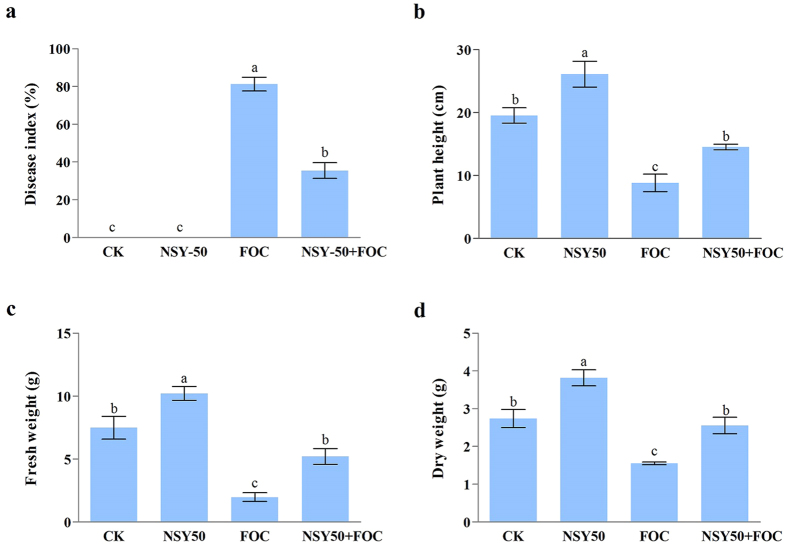
Effects of the different treatments (CK, untreated plants (control); NSY50, plants challenged with NSY50 (2.5 × 10^8^ CFU/mL); FOC, plants challenged with FOC (1 × 10^8^ CFU/mL); NSY50 + FOC: plants challenged with NSY50 for 3 days, and then with FOC) on the incidence of *Fusarium* wilt (**a**), on the various growth indices, including the height (**b**), fresh weight (**c**), and dry weight (**d**)) of ‘Jinchun No. 2’ cucumber seedlings 30 days after FOC inoculation. Each histogram represents the mean ± SE of three independent biological experiments (*n* = 3). Different letters above the bars indicate statistically significant differences by Duncan’s test (*P* < *0.05*).

**Figure 2:**
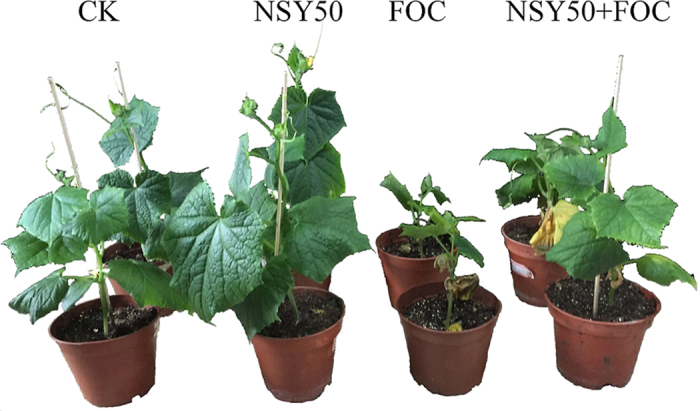
Symptom development following the different treatments (CK, untreated plants (control); NSY50, plants challenged with NSY50 (2.5 × 10^8^ CFU/mL); FOC, plants challenged with FOC (1 × 10^8^ CFU/mL); NSY50 + FOC: plants challenged with NSY50 for 3 days and then with FOC) on cucumber growth on the 30th day after FOC inoculation. The pictures are representative of two independent biological experiments.

The effects of NSY50 inoculation on disease suppression and plant growth were investigated in cucumber plants (Fig. 1). After inoculating with the pathogen, the cucumber plants appeared to have disease symptoms, including higher levels of leaf yellowing and charcoal rot, with the FOC treatment (Fig. 2). The disease incidence reached 81.25% (Fig. 1a). However, pretreatment with NSY50 significantly reduced disease occurrence, with less yellowing present in the cucumber leaves. In addition, the plant height (Fig. 1b), fresh weight (Fig. 1c) and dry weight (Fig. 1d) all responded positively to the NSY50 treatment, and inoculation with NSY50 enhanced cucumber growth. Compared to the control treatment (CK), NSY50 treatment dramatically increased the plant height by 33.62%, the fresh weight by 36.45%, and the dry weight by 39.42%. Furthermore, the growth indices of cucumbers grown with NSY50 + FOC increased significantly in comparison with those grown with FOC alone.

### Soil physico-chemical properties and enzyme activities

To determine whether NSY50 could effectively change the soil physico-chemical properties and produce better conditions, we tested the pH, C_org_, C_mic_, C_mic_/C_org_, R_mic_, qCO_2_, total P and total N. As shown in Table 1, the C_org_ and C_mic_ increased the most following NSY50 treatment, and their values were 16.95% and 23.91% higher, respectively, than those of the control treatment (CK) condition. In addition, compared to the NSY50 + FOC treatment, the FOC treatment significantly enhanced the content of qCO_2_ but reduced C_org_, C_mic_, C_mic_/C_org_, R_mic_, total P and total N. The highest reduction could be seen for C_mic_, with a decrease of 33.50%.

In this study, we evaluated seven soil enzyme activities, including catalase, invertase, urease, proteinase, phosphatase, β-glucosidase and FDA hydrolase (Table 2). In those treatments including challenge with NSY50, the activities of these seven enzymes improved to different degrees. Specifically, compared to the control treatment (CK), NSY50 application significantly increased the activities of urease, phosphatase and β-glucosidase by 115.39%, 73.65% and 161.22%, respectively. Moreover, the NSY50 + FOC treatment stimulated the activities of urease and β-glucosidase 2.25- and 2.64-fold compared with the FOC treatment alone.

**Figure 3:**
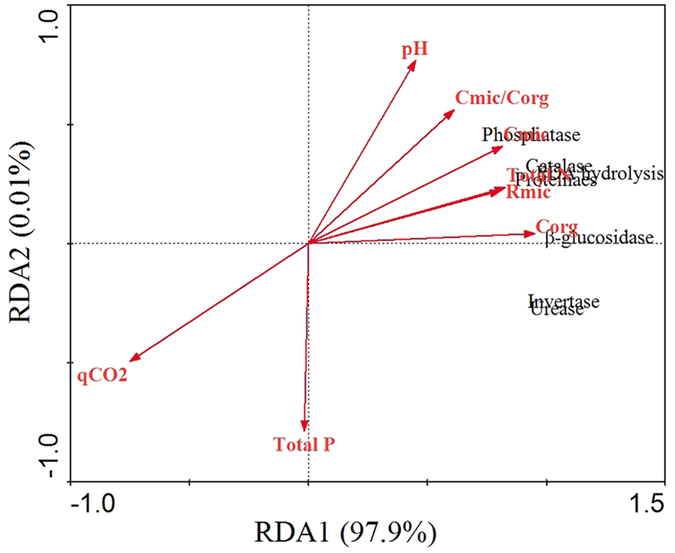
Redundancy analysis (RDA) of potential soil enzyme activities constrained by soil physicochemical and biochemical properties for soil samples collected from the four treatments (CK, untreated plants (control); NSY50, plants challenged with NSY50 (2.5 × 10^8^ CFU/mL); FOC, plants challenged with FOC (1 × 10^8^ CFU/mL); NSY50 + FOC: plants challenged with NSY50 for 3 days and then with FOC). The red vectors represent soil potential enzyme activities, and the black vectors represent soil physic-chemical properties. The following abbreviations are used: Urea-, Urease; Inve-, Invertase; β-glu, β-glucosidase; FDA-, FDA hydrolysis; Cata-, Catalase; Prot-, Proteinase; Phos-, Phosphatase.

When the soil physico-chemical properties were used to constrain the ordination of the activities of all seven enzymes in RDA (Fig. 3), the model accounted for 98% of the total variation. Based on the Monte Carlo permutation test, all explanatory variables retained in the model were significant (*P* < *0.05*) in constraining the enzyme activities. As shown in Fig. 3, amongst the measured soil physico-chemical factors, the content of C_mic_, total N and C_org_ were all correlated with the activities of phosphatase, catalase, proteinase and FDA hydrolysis. All enzyme activities along axis 1 were positively associated with C_org_, which was more highly correlated with this axis than any other variable, as indicated by the length and direction of its vector. In addition, the activity of β-glucosidase was highly related to C_org_.

### Sequencing results and microbial diversity analysis

In this study, after quality filtering, llumina sequencing -based analysis of the soil samples generated 432,941 16S rRNA gene sequences and 452,333 fungal ITS sequences that were obtained from twelve rhizosphere soil samples, with an average of 36,078 ± 2260 sequences per soil sample for bacteria and 37,694 ± 2531 sequences per soil sample for fungi. Based on a 97% nucleotide sequence identity between the reads, a total of 1634 operational taxonomic units (OTUs) for bacteria and 365 OTUs for fungi were identified (See Supplementary Fig. S1). Rarefaction curve analysis at 3% dissimilarity levels for the soil samples showed that the curves started to plateau, implying that the sampling was sufficient and reasonable.

Moreover, we estimated the Chao 1 and ACE indices for community richness and the Shannon index for community diversity (Table 3). The results showed that the Chao 1 value and ACE and Shannon indices for bacteria in the NSY50 + FOC-treated soil were significantly higher than those in the FOC-treated soil. However, for fungi, the Chao 1 and ACE indices showed the opposite results.

### Community structure and PCA analysis

**Figure 4:**
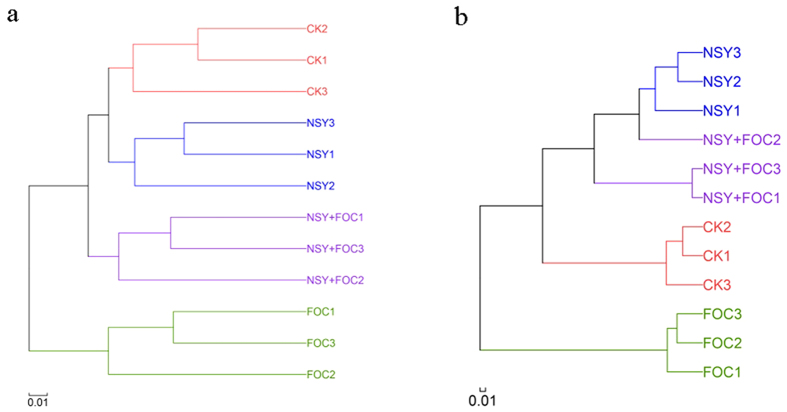
Hierarchical cluster tree constructed based on a distance matrix calculated using the unweighted UniFrac algorithm for the soil samples collected from the four treatment conditions (CK, untreated plants (control); NSY50, plants challenged with NSY50 (2.5 × 10^8^ CFU/mL); FOC, plants challenged with FOC (1 × 10^8^ CFU/mL); NSY50 + FOC: plants challenged with NSY50 for 3 days and then with FOC) 30 days after FOC inoculation. (**a**) bacteria; (**b**) fungi. *Note:* the different numbers (1, 2, 3) after the letters for all treatments indicate the three replicates. The letters NSY in the graph replace NSY50 to avoid confusion.

Hierarchical clustering analysis, calculated using the unweighted UniFrac algorithm, showed that the community structures of the bacteria and fungi collected from the soils receiving the same treatment were clustered together (Fig. 4). Soil pre-treated with NSY50 and soil treated with FOC were clearly separated from each other. In addition, PCA showed that the four treatments affected the communities differently (See Supplementary Fig. S2). Of the total variance in the data set, the first two principal components together explained 75.46% and 98.14% of the total bacterial and fungal community, respectively. In bacteria, the first principal component (PC1) was the most important, accounting for 58.26% of the total variation. The community structure may be largely responsible for these changes. Overall, PERMANOVA revealed significant differences for all OTUs according to treatment (bacteria, R^2^ = 0.6403, *P* = 0.001; fungi, R^2^ = 0.9536, *P* = 0.001).

### Effects of NSY50 challenge on soil microbial community composition

**Figure 5:**
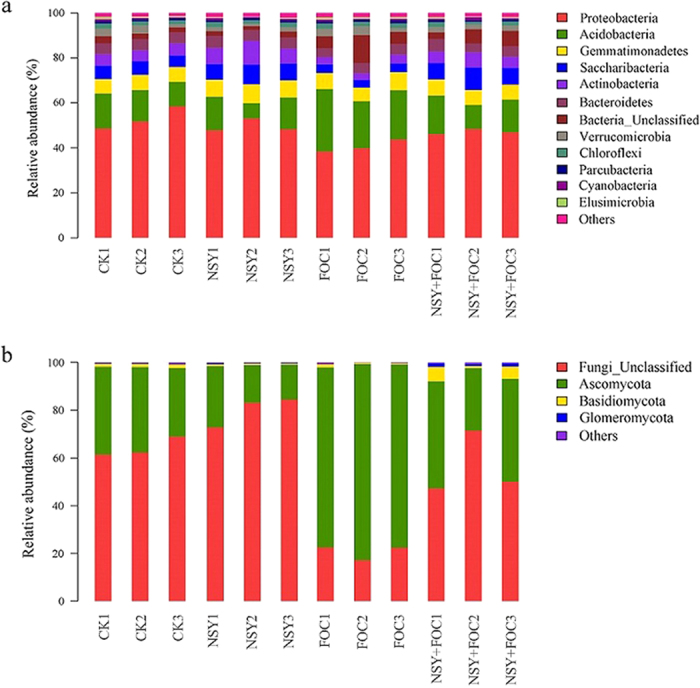
The relative abundance of the dominant bacterial phyla (**a**) and fungal phyla (**b**) for soil samples collected from the four treatment conditions (CK, untreated plants (control); NSY50, plants challenged with NSY50 (2.5 × 10^8^ CFU/mL); FOC, plants challenged with FOC (1 × 10^8^ CFU/mL); NSY50 + FOC: plants challenged with NSY50 for 3 days and then with FOC). The relative abundance was based on the proportional frequencies of those DNA sequences that could be classified at the phylum level. *Note:* the different numbers (1, 2, 3) after the letters of all treatments indicate the three replications, and the letters NSY in the graph replace NSY50 to avoid confusion.

At the phylum level, 11 bacterial and 3 fungal phyla were identified in samples from different treatments (Fig. 5). The *Firmicutes* phylum was detected in our samples, but it was not amongst the most abundant bacterial phyla shown in Fig. 5. The majority of the sequences obtained across all samples amongst the classified bacterial phyla belonged to *Proteobacteria, Acidobacteria* and *Gemmatimonadetes* (Fig. 5a). The abundance of *Saccharibacteria* and *Actinobacteria* was significantly increased in the NSY50 + FOC condition compared with the FOC condition. After the plants were inoculated (FOC and NSY50 + FOC), the relative abundance of *Acidobacteria* increased, accounting for 20% of the total population. Of the classified fungal phyla (Fig. 5b), The unclassified fungi had occupied a large proportion in different treatments, specifically in the control and NSY50 treated soil. Maybe, it was related to the used substrate, in our pot trial, we used a mixed substrate (2:1, *v/v*, peat and vermiculite). However, FOC challenge influenced the fungi composition, reducing the proportion of the unclassified fungi. In Fig. 5b, *Ascomycota, Basidiomycota* and *Glomeromycota* were identified. Interestingly, the abundance of *Basidiomycota* and *Glomeromycota* increased significantly after NSY50 treatment. The relative abundance of *Ascomycota* was the highest in the FOC condition, at nearly 80%.

**Figure 6:**
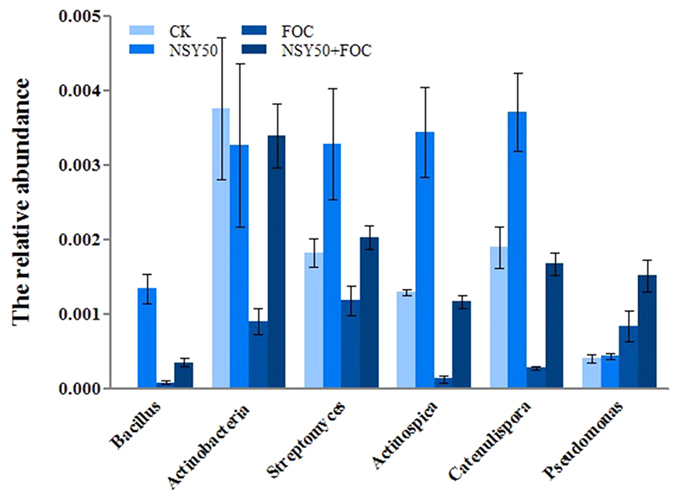
The relative abundance of the main bacterial genera for the soil samples collected from the four treatment groups (CK, untreated plants (control); NSY50, plants challenged with NSY50 (2.5 × 10^8^ CFU/mL); FOC, plants challenged with FOC (1 × 10^8^ CFU/mL); NSY50 + FOC: plants challenged with NSY50 for 3 days and then with FOC). Each histogram represents the mean ± SE of three independent biological experiments (*n* = 3).

**Figure 7:**
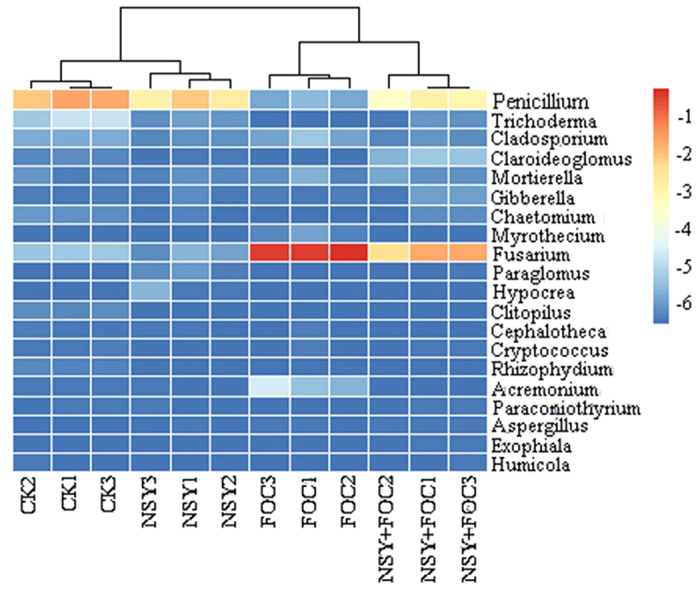
Heatmap of the top 20 classified fungal genera in soil samples collected from the four treatments (CK, untreated plants (control); NSY50, plants challenged with NSY50 (2.5 × 10^8^ CFU/mL); FOC, plants challenged with FOC (1 × 10^8^ CFU/mL); NSY50 + FOC: plants challenged with NSY50 for 3 days and then with FOC). The colour gradient from *blue* to *red* indicates an increasing relative abundance. *Note:* different numbers (1, 2, 3) after the treatments indicate the three replicates. NSY in the graph replaces NSY50 to avoid confusion.

At the genus level (Figs 6 and 7), an analysis of the relative abundance of the top 80 classified bacterial genera and the top 20 classified fungal genera using a hierarchical heat map revealed a significant difference in the FOC and NSY50-challenged treatments. Amongst the top 80 classified bacterial genera (See Supplementary Fig. S3), many BCAs had been previously identified. We chose 6 BCAs as representatives (Fig. 6), including *Bacillus, Actinobacteria, Streptomyces, Actinospica, Catenulispora*, and *Pseudomonas*. Compared with the FOC treatment, NSY50 treatment significantly enriched the relative abundance of the BCAs. The relative abundance of the top 20 classified fungal genera (Fig. 7) was not sufficiently enriched in comparison with the bacterial genera. Interestingly, only *Fusarium* was represented as an abundant genus in the FOC and NSY50 + FOC conditions. Moreover, compared to the FOC treatment, NSY50 pretreatment (NSY50 + FOC) significantly reduced the abundance of *Fusarium*, which causes cucumber *Fusarium* wilt. However, the abundance of *Penicillium* was enriched in association with NSY50 (Fig. 7), because at the species level, the unclassified *Penicillium* occupied a large proportion in NSY50 (See Supplementary Table S2).

### Correlation between soil environmental variables and abundant phyla

**Figure 8:**
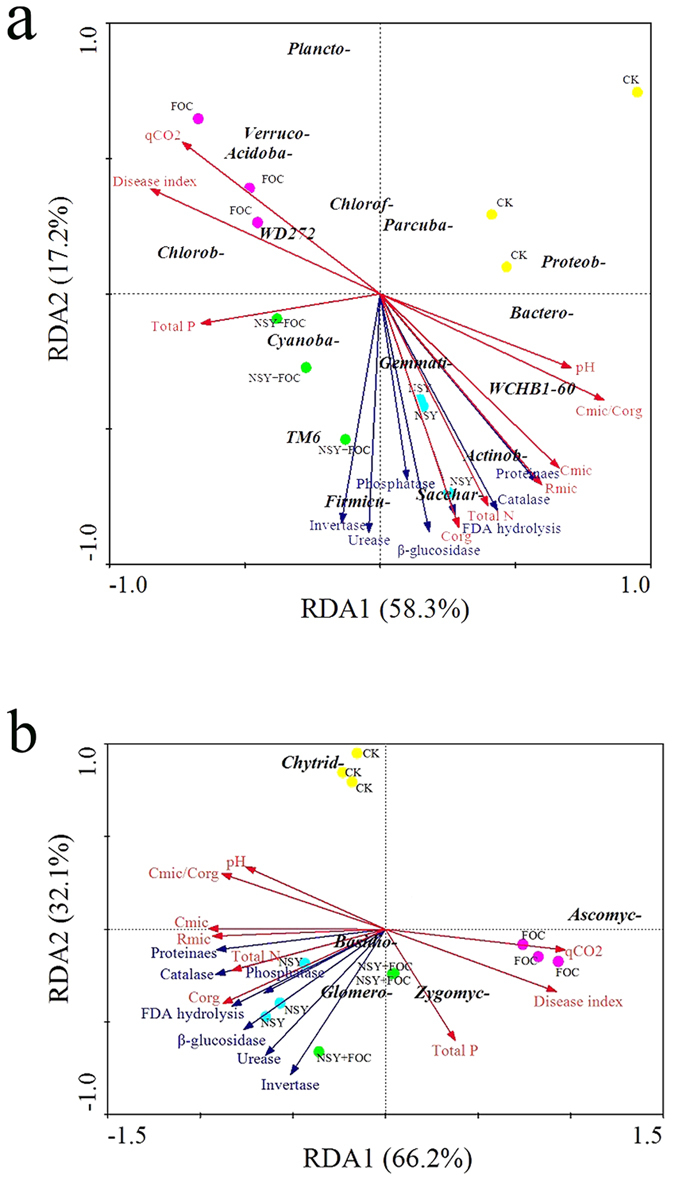
Redundancy analysis (RDA) based on the OTUs (bacterial phyla (**a**) and fungal phyla (**b**)) and selected environmental variables for soil samples collected from the four treatment conditions (CK, untreated plants (control); NSY50, plants challenged with NSY50 (2.5 × 10^8^ CFU/mL); FOC, plants challenged with FOC (1 × 10^8^ CFU/mL); NSY50 + FOC: plants challenged with NSY50 for 3 days, and then with FOC). Vectors represent selected environmental factors. The following abbreviations are used in (**a**) *Plancto*-, *Planctomycetes*; *Verruco*-, *Verrucomicrobia*; *Acidoba*- *Acidobacteria*; *Chlorob*-, *Chlorobi*; *Cyanoba*-, *Cyanobacteria*; *Gemmati-, Gemmatimonadetes*; *Firmicu*-, *Firmicutes*; *Sacchar*-, *Saccharibacteria*; *Actinob*-, *Actinobacteria*; *Bactero*- *Bacteroidetes*; *Proteob*-, *Proteobacteria*; *Chlorof*-, *Chloroflexi*; *Parcuba*-, *Parcubacteria*. The following abbreviations are used in (**b**): *Chytrid*-, *Chytridiomycota*; *Basidio*-, *Basidiomycota*; *Glomero*-, *Glomeromycota*; *Zygomyc*-, *Zygomycota*; *Ascomyc*-, *Ascomycota. Note*: 99.8% of ‘fungal’ sequences (including the Unclassified fungi) were actually fungal.

Microflora composition is closely related to the overall ecological environment of the soil; environmental characteristics, such as the soil enzyme activity and physico-chemical properties, affect soil microbes and ecosystem function and can be manipulated to optimally inhibit *Fusarium* wilt. Correlations based on phyla were analysed (Fig. 8).

RDA analysis showed that the full RDA model accounted for 75.5% of the total bacterial variation (Fig. 8a). The first component (RDA1) explained 58.3% of the total variation in the bacterial phyla, whilst the second component (RDA2), explained 17.2%. The NSY50 bacterial communities were associated with higher soil pH, C_mic_, R_mic_, total N, C_org_ and C_mic_/C_org_, and the bacterial communities associated with FOC treatment were highly correlated with qCO_2_ and disease. All soil enzyme activities had a significant effect on the soil bacterial communities in the NSY50 and NSY50 + FOC conditions. In addition, the phyla *Gemmatimonadetes, Actinobacteria, Saccharibacteria*, and *Firmicutes* were strongly associated with the enzyme activities and the total content of N, C_org_, C_mic_ and R_mic_. For the fungal phyla (Fig. 8b), the first two axes of the RDA explained 66.1% and 32.1% of the total variation. The abundance of *Glomeromycota* and *Basidiomycota* was positively correlated with all soil enzyme activities, and the abundance of *Zygomycota* was significantly related to the total P content.

## Discussion

The rhizosphere microbiome in different conditions varies in diversity and in the response to soil environmental changes; moreover, they are essential for controlling plant diseases and, consequently, plant health. However, how the rhizosphere microbiome modulates disease suppression remains an open question. In this study, we assessed the positive effects of NSY50 challenge on the suppression of cucumber *Fusarium* wilt. Many previous findings have demonstrated that the addition of BCAs can effectively control soil-borne diseases, especially *Fusarium* wilt^35^, and can lead to a broad-range suppressive effect^36^,^37^. In addition, upon attack by a fungal root pathogen, plants can mobilize their unique rhizospheric microbial populations for protection^38^,^39^. Thus, we explored the response of the soil microbial community to NSY50 application using Illumina sequencing (16S rRNA gene and the ITS regions) on the Miseq platform.

Our research provides insight into how NSY50 challenge influences variations in the physicochemical properties of the substrate, the enzymatic activities and rhizosphere microbiome to suppress cucumber *Fusarium* wilt. The two hypotheses were supported in the following ways. First, many other reports suggest that plants can alter their specific rhizospheric community when they were attacked by pathogens^40^,^41^. So in our study NSY50 challenge may have a selective effect on rhizospheric bacterial and fungal communities, which, in turn, changes aspects of the substrate environment. Second, FOC inoculation led to many FOC populations colonizing the rhizosphere, which may alter the composition of the rhizospheric community. Although the FOC populations include pathogenic and non-pathogenic FOC, and they could not be separately quantified^42^. In our study, the results of the pot experiment and the higher disease incidence indicated that the pathogenic FOC were a major component of the FOC assayed in the rhizospheric soil.

In the pot experiment, NSY50 application had a positive effect on the growth of cucumber plants; the plant height, fresh weight and dry weight were all increased to different degrees compared to the CK treatment. Furthermore, the disease incidence was significantly suppressed, suggesting that NSY50 has the capacity to stimulate plant growth and suppress soil-borne pathogens. These results, in combination with other reports, demonstrate that the rhizobacterium *Paenibacillus* polymyxa E681 possesses potential biocontrol ability through the production of various plant hormones, antibiotics and hydrolytic enzymes that promote the immunity of the rhizosphere and inhibit plant pathogens^43^.

Interestingly, the observation of richness (the ACE and Chao 1 indices) and more diverse bacterial communities in the NSY50 + FOC treatment condition in comparison with the FOC treatment condition, which are in line with many previous reports showing that greater bacterial diversity in suppressive soils promotes resistance to *Fusarium* wilt of cucumber^44^. Furthermore, microbial community composition and structural analysis showed that a high relative abundance of *Bacillus, Actinobacteria, Streptomyces, Actinospica, Catenulispora* and *Pseudomonas* genera were identified in the NSY50 challenged soil, and compared to the FOC treatment, their relative abundance was significantly increased with the NSY50 + FOC treatment. Indeed, previous studies have suggested that the *Pseudomonas, Bacillus* and *Actinomycetes* genera are dominant BCAs in agricultural soils and effectively control different soil-borne pathogens^45^,^46^,^47^. Specially, the genera *Bacillus* and *Pseudomonas* are well-known BCAs and have been frequently reported to be responsible for suppressing soil-borne pathogens and stimulating plant growth by producing antifungal compounds and bioactive metabolites, synthesizing various hormones, utilizing root exudates, and limiting competition with other microorganisms^48^,^49^. In addition, the genera *Bacillus and Pseudomonas* belong to the phyla *Firmicutes*, which includes many potential BCAs. Rosenzweig *et al*.^50^ and Mendes *et al*.^38^ have demonstrated that the abundance of the phyla *Firmicutes* is high in suppressive soils. Other potentially beneficial genera, including *Actinobacteria, Streptomyces, Actinospica* and *Catenulispora,* belong to *Actinobacteria* phylum, which has been identified as a dominant phylum in NSY50 + FOC-treated soil. This result is consistent with the findings of a previous study showing that *Actinobacteria* is the most abundant phylum in disease-suppressive soils^20^. Therefore, our results, combined with previous research, suggest that NSY50 may play a key role in the soil environment that influences the abundance of the genera *Bacillus, Actinobacteria, Streptomyces, Actinospica, Catenulispora* and *Pseudomonas,* which are involved in the suppression of cucumber *Fusarium* wilt. However, whether this mechanism is direct or indirect is currently unknown and will be the subject of future research.

The use of NSY50 in the suppression of cucumber *Fusarium* wilt occurs not only through shifting the rhizospheric bacterial community but also through altering the composition of the fungal community. In this study, *Ascomycota, Basidiomycota* and *Glomeromycota* were found in all rhizosphere samples, and *Ascomycota* was the most abundant phylum amongst all fungal sequences. This was consistent with the results of previous reports showing that *Ascomycota* was also the most abundant fungal phylum^51^. However, NSY50 application significantly reduced the abundance of the *Ascomycota* phylum. Sprague *et al*.^52^ and Câmara *et al*.^53^ demonstrated that the *Ascomycota* phylum included some fungal pathogens. Regarding the fungal genera, remarkably, NSY50 + FOC treatment significantly decreased the abundance of *Fusarium*, which causes cucumber *Fusarium* wilt, indicating that NSY50 application can be considered to be an effective strategy to control cucumber *Fusarium* wilt.

RDA analysis revealed a correlation between the soil environmental variables and the bacterial and fungal phyla present. The soil total P was detected at a higher level in the NSY50 + FOC soil compared to the FOC soil. Furthermore, the disease index was highly correlated with the FOC treatment. This result was similar to that of McNeill *et al*.^54^ who found that a lower wheat *Rhizoctonia* root rot disease index may be related to a higher P content, which could improve resistance to plant pathogens. Higher pH, C_mic_/_Corg_, C_mic_, C_org_, R_mic_, total N and all soil enzyme activities were noted in the NSY50 treatment condition, indicating a negative correlation with the disease index. In contrast, the *Acidobacteria* phylum had a positive relationship with the disease index. However, our results did not agree with those of a previous study showing higher pH and the presence of the *Acidobacteria* phylum in disease-suppressive soil^55^. This may be because the application of NSY50 and FOC produce a wide range of responses to affect the soil environment, such as altering enzyme activities, phy-chemical properties and other microbial communities, to maintain an optimal environment to suppress disease. Increasing evidence has indicated that shifts in soil properties, including enzyme activities and phy-chemical properties, may affect the microbial community, thus influencing the interaction between plants and beneficial microbes. However, the mechanisms of this complex interaction between soil properties and the microbial community need further investigation.

To our knowledge, this is the first research to use high-throughput sequencing to study the interaction between cucumber and the beneficial microbe, NSY50. NSY50 application may systemically affect plant-soil interactions by increasing soil richness and the diversity and the abundance of certain BCAs, such as the genera *Bacillus, Actinobacteria, Streptomyces, Actinospica, Catenulispora* and *Pseudomonas.* These genera are known to have an antagonistic action against pathogens^14^,^19^,^20^,^56^,^57^, thus reducing cucumber *Fusarium* wilt and stimulating cucumber growth. Furthermore, the rhizospheres of cucumber plants subjected to NSY50 + FOC treatment showed a lower abundance of *Fusarium* than the rhizospheres of cucumber plants grown with FOC. Several soil properties, such as enzyme activities, pH, C_mic_/C_org_, C_mic_, C_org_, R_mic_ and total N, may be involved in this process in the optimal suppression state. Therefore, our results suggest that NSY50 application changes the soil enzyme activities, phy-chemical properties and microbial community structure, thus increasing the beneficial microbes and reducing the pathogenic fungi. Ultimately, this produces a condition of disease suppression, thereby leading to a reduction in cucumber *Fusarium* wilt.

## Materials and Methods

### Microbial culture conditions

*Paenibacillus polymyxa*-NSY50 was used in this study. Bacteria were grown on LB medium (10 g/L tryptone, 10 g/L NaCl, 5 g yeast extract). For preparation of the bacterial inoculum, NSY50 was grown in liquid LB medium (10 g/L tryptone, 10 g/L NaCl and 5 g yeast extract per litre of distilled water) at 28 °C for 72 h on a rotary shaker (180 rpm) and then centrifuged at 6000 g for 5 min to collect the cells. The harvested cells were re-suspended in distilled water to generate 2.5 × 10^8^ colony-forming units (CFU)/mL for inoculation.

The cucumber *Fusarium* wilt pathogen *Fusarium oxysporum* f. sp. *cucumerinum* (FOC) was originally isolated from susceptible cucumber plants and stored at 4 °C in potato dextrose agar (PDA) until use. In our previous work, it was demonstrated to have strong pathogenicity.

### Pot experimental design

The pot trial was conducted by using a light growth chamber in Nanjing Agricultural University. We used ‘Jinchun No. 2’, a disease-susceptible cucumber cultivar, in this experiment. For pre-germination, seeds were surface-sterilized in ethanol (70%) for 30 s, followed by 20 min of shaking in a 2% sodium hypochlorite solution. After sterilization, the seeds were repeatedly washed in sterile distilled water, after which germination was completed on moist filter paper in the dark at 28 °C for 24 h. The germinated seeds were sown in plastic pots (diameter 10 cm) that contained a mixed substrate (2:1, *v/v*, peat and vermiculite) and maintained in the light growth chamber at 28 °C in the daytime, 18 °C at night with a 16 h light/8 h dark cycle and a relative humidity of 75%. Seedlings with two true leaves were inoculated with 2.5 × 10^8^ CFU/mL.

After full development of the second leaf, the seedlings were subjected to inoculation treatment. The assays included four treatments: (1) CK, untreated plants (control), (2) NSY50, plants challenged with NSY50, (3) FOC, plants challenged with FOC, and 4) NSY50 + FOC: plants challenged with NSY50 for 3 days and then with FOC. For the NSY50 pretreatment, 20 mL of the NSY50 cell suspension (2.5 × 10^8^ CFU/mL) was applied as a root drench 3 days prior to pathogen infection. The pathogen concentration was 1 × 10^8^ CFU/mL, and pathogens were administered by the root-irrigation method. Each pot had a root volume of 20 mL, and the rhizosphere soil was directly irrigated. The control treatments consisted of an equivalent volume of sterile distilled water, and the whole growth process was supplied with half-strength Hoagland’s nutrient solution every 7 days. Pots of seedlings were arranged in a completely randomized block design with three replications for each treatment. Only one plant was planted per pot, resulting in a total of 96 seedlings for the four treatments (24 seedlings per treatment).

Disease incidence was assessed 30 days after inoculation with FOC as described previously. In brief, disease incidence was rated using an index of 0 to 4 (0 = healthy; 1 = less than 25% of leaves wilted; 2 = 25% to 50% of leaves wilted; 3 = 50% to 75% of leaves wilted; 4 = 75% to 100% of leaves wilted)^58^. The disease severity index (DSI) was then calculated using the formula: Disease index = [∑(rating × number of plants rated)/(total number of plants × highest rating)] × 100.

### Growth analysis

Plant height (cm), fresh weight (g) and dry weight (g) were recorded on the 30^th^ day of the inoculation of FOC. Three seedlings were randomly selected. In brief, the fresh weight was measured on an electronic scale after a sterile distilled water wash. The dry weight was subsequently obtained by drying the plant samples at 105 °C for 15 min, and the temperature was then reduced to 75 °C until the weight remained constant.

### Analysis of soil physical and chemical properties

Soil samples were obtained from the three biological replicate pots, and the rhizosphere soil samples were collected on the 30^th^ day after challenge with FOC. All collected samples were sieved (2 mm) and separated equally into two subsamples^59^. One portion of each sample was frozen at −80 °C for DNA extraction, and the other portion was used for the determination of the soil microbial and biochemical parameters.

The soil pH was determined with a glass electrode (soil/distilled water = 1:5, *w/v*). The total organic carbon (C_org_) was determined by dichromate oxidation, and the microbial biomass carbon (C_mic_) was determined by the chloroform fumigation–extraction method^60^,^61^. Basal respiration (R_mic_) was determined by measuring CO_2_ evolution. Briefly, an equivalent to 10 g distilled water of fresh sample mixed with 60 mg glucose was incubated in 250 mL airtight glass vessels at 25 °C for 6 h. The CO_2_ produced from the sample was absorbed in 0.05 M NaOH and determined by titration with HCl^62^. The metabolic quotient (qCO_2_) was defined as the ratio of R_mic_ to C_mic_^63^. The micro-Kjeldahl method was used to determine total N^64^. The total P was determined using the molybdenum-antimony anti-spectrophotometric method.

### Soil enzyme activity analysis

Urease activity in the soil was measured by spectrophotometry at 578 nm as the NH_4_–N released from 5.0 g of soil after a 24-h incubation period at 37 °C with 10% (*w/v*) urea solution in 20 mL of citrate buffer at pH 6.7 65. Catalase activity was determined by back-titration of residual H_2_O_2_ in the soil with 0.1 M KMnO_4_ in the presence of H_2_SO_4_ 66. Neutral phosphatase activity was measured with a spectrophotometer at 570 nm, as described by Wu *et al*.^67^. Proteinase activity was carried out by the colorimetric method using ninhydrin at a wavelength at 500 nm^66^. Invertase activity was measured spectrophotometrically using the 3,5-dinitrosalicylic acid method of Hou *et al*.^68^. The determination of the activity of β-glucosidase and hydrolysis of fluorescein diacetate (FDA) was performed following a procedure described by Dick *et al*.^69^ and Adam and Duncan^70^ with some modifications.

### Soil DNA extraction, PCR and sequencing

Soil DNA was extracted from 0.5 g of fresh soil using a FastDNA^TM^ SPIN Kit (MP Biomedicals, Santa Ana, CA, USA) according to the manufacturer’s instructions. The concentration and quality of the extracted DNA were quantified with an Eppendorf Biophotometer plus (Eppendorf, Germany), and the DNA was stored at −20 °C.

For each sample, we amplified the V4-V5 region of the bacterial 16S rRNA gene using a broadly conserved primer set (515F-907R) and the fungal ITS sequence (ITS1-ITS2). The primer set 515 F (forward primer: 5′-GTGCCAGCMGCCGCGG-3′) and 907 R (reverse primer: 5′-CCGTCAATTCMTTTRAGTTT-3′ 71) was used for bacterial ITS sequence amplification, whilst the primer set ITS1 (forward primer: 5′-CTTGGTCATTTAGAGGAAGTAA-3′) and ITS2 (reverse primer: 5′-GCTGCGTTCATCGATGC-3′) was used for fungal ITS sequence amplification^72^. The PCR reactions were carried out in a 20 μL reaction mixture containing 0.8 μL of each primer, 2 μL dNTPs (2.5 mM), 4 μL of 5 × FastPfu Buffer, 0.4 μL of FastPfu polymerase and 10 ng of soil DNA template^73^. Amplification was initiated at 95 °C for 5 min, followed by 27 (bacteria) or 29 (fungi) cycles of denaturation at 95 °C for 30 s, annealing at 55 °C for 30 s, and extension at 72 °C for 45 s, followed by a final elongation at 72 °C for 10 min. The PCR products were pooled and visualized on 2% agarose gels, purified using an AxyPrep^TM^ DNA Gel Extraction Kit (Axygen Biosciences; Union City, CA, USA) according to the manufacturer’s instructions, and quantified using QuantiFluor^TM^-ST (Promega, US).

High-throughput sequencing was carried out on the Illumina MiSeq platform by Shanghai Biozeron Bio-pharm Technology Co., Ltd (Shanghai, China). After pyrosequencing, the raw 16S rRNA gene and ITS pyrosequencing data were demultiplexed and quality-filtered using QIIME (version 1.17) based on criteria described in Supplementary Table S1. Finally, using UPARSE (version 7.1 http://drive5.com/uparse/) with a cut-off of 97% similarity, OTUs were clustered, and chimeric sequences were identified and removed using UCHIME. The phylogenetic affiliation of each 16S rRNA gene sequence was analysed using the RDP Classifier (http://rdp.cme.msu.edu/) against the SILVA (SSU123)16S rRNA database with a confidence threshold of 70% 74. The ITS sequencing data was classified by using the Unite (Release 6.0 http://unite.ut.ee/index.php).

### Statistical analyses

All data were statistically analysed using the SPSS 20.0 program (SPSS Inc., Chicago, IL, USA), and significance was assigned at *P* < *0.05* using one-way analysis of variance (ANOVA) with Duncan’s tests. Permutational multivariate analysis of variance (PERMANOVA) was performed to evaluate significant differences in the microbial community composition according to the different treatments. For the diversity analysis, a rarefaction curve was generated to compare the relative levels of bacterial and fungal OTU diversity across all soil samples using Mothur software^75^. Hierarchical cluster trees based on subsample files were calculated by the unweighted UniFrac algorithm^76^. Redundancy analysis (RDA) using Canoco version 4.5 (Biometry, Wageningen, The Netherlands) and heat maps based on the retained OTUs were created using R (version 3.0.2) with the gplots package.

## Additional Information

**How to cite this article:** Shi, L. *et al. Paenibacillus polymyxa* NSY50 suppresses *Fusarium* wilt in cucumbers by regulating the rhizospheric microbial community. *Sci. Rep.* **7**, 41234; doi: 10.1038/srep41234 (2017).

**Publisher's note:** Springer Nature remains neutral with regard to jurisdictional claims in published maps and institutional affiliations.

## Supplementary Material

Supplementary Material

## Figures and Tables

**Table 1:** Effects of NSY50 and FOC challenges on the physicochemical and biochemical properties of the growth media in cucumber rhizospheric soil on the 30^th^ day after challenge with FOC.

Treatment	pH (H_2_0)	C_org_ (g kg^−1^)	C_mic_ (μg g^−1^)	C_mic_/C_org_ (%)	R_mic_ (μg CO_2_ g^−1^ h^−1^)	qCO_2_ (μg CO_2_ C mg^−1^ C_mic_ h^−1^)	Total P (mg kg^−1^)	Total N (g kg^−1^)
CK	6.27 ± 0.06a	17.23 ± 0.26c	547.91 ± 8.29b	3.18 ± 0.01b	4.99 ± 0.07b	9.12 ± 0.11c	295 ± 48c	0.53 ± 0.00b
NSY50	6.31 ± 0.03a	20.15 ± 0.27a	678.94 ± 8.95a	3.37 ± 0.05a	5.41 ± 0.10a	7.97 ± 0.13d	342 ± 21c	0.61 ± 0.02a
FOC	5.97 ± 0.05b	15.36 ± 0.18d	328.78 ± 3.80d	2.14 ± 0.03d	4.14 ± 0.06c	12.58 ± 0.05a	500 ± 19b	0.46 ± 0.04c
NSY50 + FOC	6.04 ± 0.02b	19.02 ± 0.14b	494.43 ± 3.61c	2.60 ± 0.04c	5.01 ± 0.08b	10.13 ± 0.21b	636 ± 17a	0.56 ± 0.00ab

*Note*: The four treatments were: (1) CK, untreated plants (control); (2) NSY50, plants challenged with NSY50 (2.5 × 10^8^ CFU/mL); (3) FOC, plants challenged with FOC (1 × 10^8^ CFU/mL); (4) NSY50 + FOC: plants challenged with NSY50 for 3 days and then with FOC. C_org_, total organic carbon, C_mic_, microbial biomass carbon; R_mic_, basal respiration, qCO_2_, metabolic quotient. Data represent the mean ± standard deviation of three replicates. Different letters in each column indicate statistically significant differences according to Duncan’s test (*P* < *0.05*).

**Table 2:** The effect of NSY50 and FOC challenges on the enzyme activities of cucumber rhizospheric soil on the 30^th^ day after FOC challenge.

Treatment	Catalase (mL(0.1 M K_2_MnO_4_) g^−1^ h^−1^)	Invertase (mg glucose g^−1^ h^−1^)	Urease (mg NH_4_^+^-N kg^−1^ h^−1^)	Proteinase (mg glycine kg^−1^ h^−1^)	Phosphatase (mg phenol g^−1^ h^−1^)	β-glucosidase (μg hydrolysed p-nitrophenol g^−1^ h^−1^)	FDA hydrolysis (μg FDA g^−1^ h^−1^)
CK	3.65 ± 0.02c	22.85 ± 0.27d	15.2 ± 0.20b	8.89 ± 0.44b	1.67 ± 0.93ab	236.77 ± 13.40c	54.70 ± 1.61c
NSY50	4.30 ± 0.01a	33.14 ± 0.12a	32.74 ± 0.44a	11.23 ± 0.29a	2.90 ± 0.83a	618.48 ± 26.01a	85.97 ± 2.31a
FOC	2.98 ± 0.13d	25.35 ± 0.42c	14.34 ± 0.03b	5.93 ± 0.51c	1.14 ± 0.46b	165.72 ± 17.34d	42.93 ± 2.95d
NSY50 + FOC	3.98 ± 0.02b	29.11 ± 0.37b	32.30 ± 0.47a	9.41 ± 0.14b	2.24 ± 0.38ab	438.45 ± 24.55b	67.70 ± 2.00b

*Note*: Different letters in each column indicate statistically significant differences by Duncan’s test (*P* <* 0.05*). The four treatments are: (1) CK, untreated plants (control); (2) NSY50, plants challenged with NSY50 (2.5 × 10^8^ CFU/mL); (3) FOC, plants challenged with FOC (1 × 10^8^ CFU/mL); (4) NSY50 + FOC: plants challenged with NSY50 for 3 days, then with FOC.

**Table 3:** The mean of the Chao 1, ACE and Shannon indices of rhizosphere soil challenged with NSY50 and FOC at 97% similarity.

Treatment	Community characteristics					
	Bacterial community			Fungal community		
CK	ACE	Chao 1	Shannon	ACE	Chao 1	Shannon
	1389b	1394a	5.53bc	206b	211b	1.80a
NSY50	1400ab	1397a	5.65a	190b	185c	1.18b
FOC	1336c	1340b	5.46c	225a	236a	1.34b
NSY50 + FOC	1431a	1429a	5.58ab	202b	207b	1.59ab

*Note*: Different letters in each column indicate statistically significant differences based on Duncan’s test (*P* < *0.05*).
