# Supplementary material for: Paenibacillus polymyxa NSY50 suppresses Fusarium wilt in cucumbers by regulating the rhizospheric microbial community
Source: Sci Rep. 2017 Feb 13;7:41234. doi: 10.1038/srep41234 (PMC5304210; doi:10.1038/srep41234)
Supplement: Supplementary Material [file srep41234-s1.pdf]

Paenibacillus polymyxa NSY50 suppresses *Fusarium* wilt in cucumbers by regulating the rhizospheric microbial community

Lu Shi[a], Nanshan Du[a], Sheng Shu[a], Jin Sun[a], Shuzhan Li[a], Shirong Guo[a*]

[a] Key Laboratory of Southern Vegetable Crop Genetic Improvement in Ministry of Agriculture, College of Horticulture, Nanjing Agricultural University, Nanjing 210095, People's Republic of China.

[*] Corresponding author: TEL./FAX: +862584395267.
E-mail address: srguo@njau.edu.cn (S.–R. Guo).

Supplementary Table 1

The criterions of choosing the raw 16S rRNA genes and ITS sequence pyrosequencing data.

	criterions
**(1)**	The 250 bp reads were truncated at any site receiving an average quality score <20 over a 10 bp sliding window, discarding the truncated reads that were shorter than 50bp;
**(2)**	exact barcode matching, 2 nucleotide mismatch in primer matching, reads containing ambiguous characters were removed;
**(3)**	only sequences that overlap longer than 10 bp were assembled according to their overlap sequence. Reads which could not be assembled were discarded.

Supplementary Table 2

The number of Penicillum spp. in different samples at the species level.

OTU ID	CK1	CK2	CK3	FOC1	FOC2	FOC3	NSY1	NSY2	NSY3	NSY+FOC1	NSY+FOC2	NSY+FOC3
Penicillium brasilianum	6	8	0	0	0	0	2	2	0	7	0	6
Penicillium citrinum	14	5	0	1	0	1	2	0	0	0	0	1
Penicillium coffeae	9	8	11	10	4	11	2	1	1	1	6	1
Penicillium mallochii	0	0	0	1	1	0	0	2	1	0	0	0
Penicillium olsonii	114	112	132	7	4	0	5	3	2	3	8	6
Penicillium oxalicum	15	3	7	2	2	2	10	1	0	2	3	1
Penicillium sumatrense	0	2	0	0	0	0	0	1	0	2	0	0
Penicillium toxicarium	1	0	0	3	0	3	0	2	0	0	0	0
Penicillium tularense	0	0	0	0	0	0	0	0	26	0	0	0
Penicillium_Unclassified	12972	11012	8678	296	220	225	7595	5905	4505	4151	3096	3487

Supplementary Figure 1.

（a）　　　　　　　　　　　　　　　　　（b）

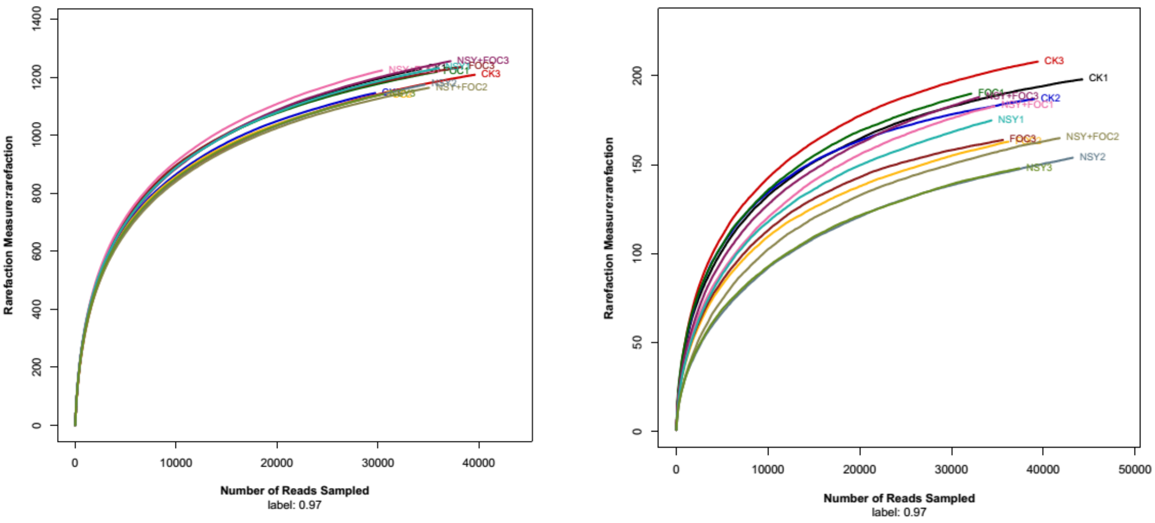

Supplementary Figure 1. Rarefaction analysis at 3% dissimilarity levels for soil samples collected from the four treatments (CK, untreated plants (control); NSY50, plants challenged with NSY50 (2.5×10^8 CFU/mL); FOC, plants challenged with FOC (1×10^8 CFU/mL); NSY50 + FOC: plants challenged with NSY50 for 3 days, and then with FOC). (a) bacterial; (b)fungi. The *vertical axis* shows the average number of OUTs that would be expected to be found after sampling the number of sequences shown on the *hoorizontal axis*.

Supplementary Figure 2.

（a）　　　　　　　　　　　　　　　　　（b）

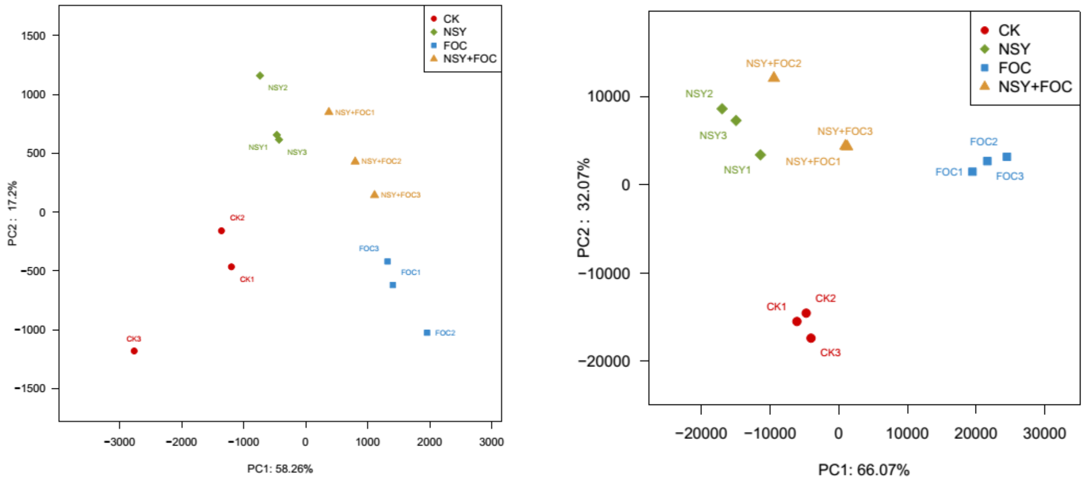

Supplementary Figure 2. Principal Component Analysis for soil samples collected from the four treatments (CK, untreated plants (control); NSY50, plants challenged with NSY50 (2.5×10^8 CFU/mL); FOC, plants challenged with FOC (1×10^8 CFU/mL); NSY50 + FOC: plants challenged with NSY50 for 3 days, and then with FOC). (a) bacteria; (b) fungi.

Supplementary Figure 3.

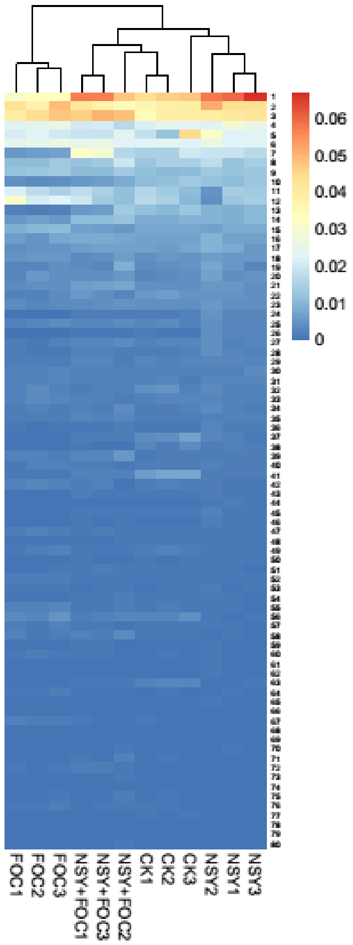

Supplementary Figure 3. Heatmap of the top 80 classified bacterial genera for soil samples collected from the four treatments (CK, untreated plants (control); NSY50, plants challenged with NSY50 (2.5×10^{8} CFU/mL); FOC, plants challenged with FOC (1×10^{8} CFU/mL); NSY50 + FOC: plants challenged with NSY50 for 3 days, and then with FOC). The color gradient from blue to red indicates increasing the relative abundance.

Supplementary Figure 4.

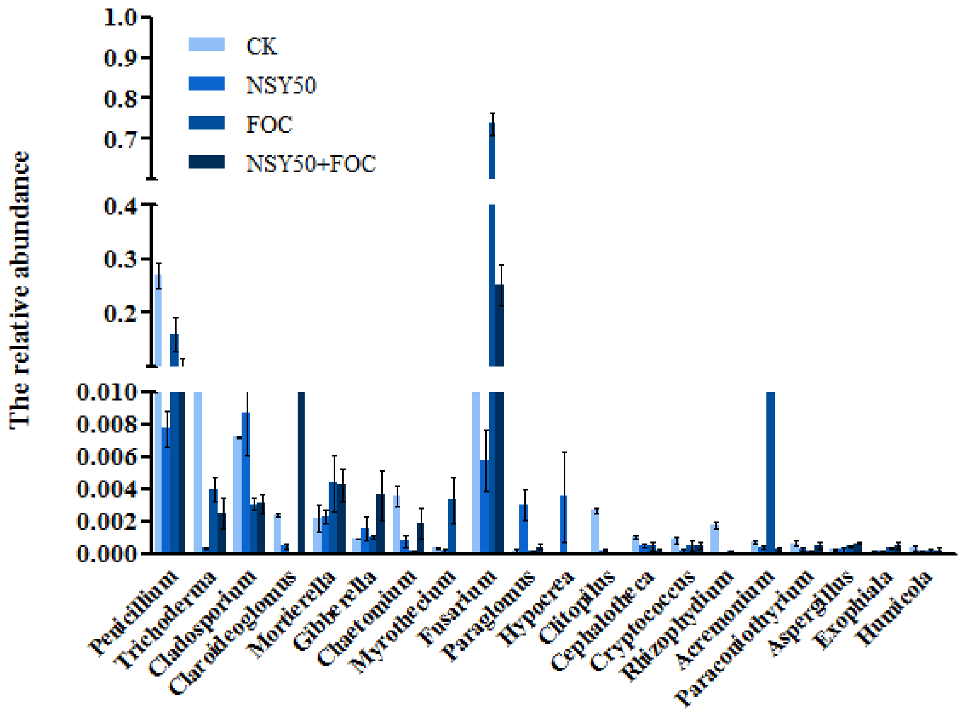

Supplementary Figure 4. The relative abundance of the main fungal genera for the soil samples collected from the four treatment groups (CK, untreated plants (control); NSY50, plants challenged with NSY50 (2.5×10^8 CFU/mL); FOC, plants challenged with FOC (1×10^8 CFU/mL); NSY50 + FOC: plants challenged with NSY50 for 3 days, and then with FOC). Each histogram represents the mean $\pm$SE of three independent biological experiments (n=3).
